# *Rhipicephalus microplus* serine protease inhibitor family: annotation, expression and functional characterisation assessment

**DOI:** 10.1186/s13071-014-0605-4

**Published:** 2015-01-07

**Authors:** Manuel Rodriguez-Valle, Tao Xu, Sebastian Kurscheid, Ala E Lew-Tabor

**Affiliations:** The University of Queensland, Queensland Alliance for Agriculture & Food Innovation, Queensland Biosciences Precinct, 306 Carmody Rd, St. Lucia Qld, 4072 Australia; Murdoch University, Centre for Comparative Genomics, Perth, Western Australia 6150 Australia; University Hospital Lausanne, Lausanne, VD Switzerland

**Keywords:** Genome, Protease inhibitor, Rhipicephalus microplus, serpin, Cattle tick

## Abstract

**Background:**

*Rhipicephalus (Boophilus) microplus* evades the host’s haemostatic system through a complex protein array secreted into tick saliva. Serine protease inhibitors (serpins) conform an important component of saliva which are represented by a large protease inhibitor family in *Ixodidae*. These secreted and non-secreted inhibitors modulate diverse and essential proteases involved in different physiological processes.

**Methods:**

The identification of *R. microplus* serpin sequences was performed through a web-based bioinformatics environment called Yabi. The database search was conducted on BmiGi V1, BmiGi V2.1, five SSH libraries, Australian tick transcriptome libraries and RmiTR V1 using bioinformatics methods. Semi quantitative PCR was carried out using different adult tissues and tick development stages. The cDNA of four identified *R. microplus* serpins were cloned and expressed in *Pichia pastoris* in order to determine biological targets of these serpins utilising protease inhibition assays.

**Results:**

A total of four out of twenty-two serpins identified in our analysis are new *R. microplus* serpins which were named as RmS-19 to RmS-22. The analyses of DNA and predicted amino acid sequences showed high conservation of the *R. microplus* serpin sequences. The expression data suggested ubiquitous expression of RmS except for RmS-6 and RmS-14 that were expressed only in nymphs and adult female ovaries, respectively. RmS-19, and -20 were expressed in all tissues samples analysed showing their important role in both parasitic and non-parasitic stages of *R. microplus* development. RmS-21 was not detected in ovaries and RmS-22 was not identified in ovary and nymph samples but were expressed in the rest of the samples analysed. A total of four expressed recombinant serpins showed protease specific inhibition for Chymotrypsin (RmS-1 and RmS-6), Chymotrypsin / Elastase (RmS-3) and Thrombin (RmS-15).

**Conclusion:**

This study constitutes an important contribution and improvement to the knowledge about the physiologic role of *R. microplus* serpins during the host-tick interaction.

## Background

Ticks are worldwide-distributed ectoparasites that have evolved as obligate haematophagous arthropods of animals and humans. These parasites have been categorised after mosquitoes as the second most important group of vectors transmitting disease-causing agents to mammals [1,2]. In particular, the cattle tick (*Rhipicephalus microplus*) is considered the most economically important ectoparasite of cattle distributed in tropical and subtropical regions of the world. The principal reason for this affirmation is that *R. microplus* affects beef and dairy cattle producers causing direct economic losses due to host parasitism and tick borne diseases such as anaplasmosis and babesiosis [3,4].

The success of the parasitic cycle of *R. microplus* begins with the larval capability to overcome haemostatic and immunological responses of the host. Following larval attachment, a great amount of blood is ingested and digested by ticks in order to complete their parasitic cycle. The full-engorged adult females drop off from host to initiate the non-parasitic phase with the laying and hatching of eggs. *R. microplus* has an intensive production and physiological secretion of proteins during the entire parasitic cycle in order to disrupt host responses such as protease inhibitors which play an important role in tick survival, feeding and development [5-8]. Serpins (*Se*rine *P*rotease *In*hibitor*s*) are important regulatory molecules with roles during host- parasite interactions such as fibrinolysis [9], host response mediated by complement proteases [10], and inflammation [11-13] among other tick physiological functions [14,15]. These protease inhibitors conformed a large superfamily that is extensively distributed within bacteria, insects, parasite, animals and plants [16,17]. Serpins differ from Kunitz protease inhibitors by distinctive conformational change during the inhibition of their target proteases. The presence of a small domain designated as the reactive center loop (RCL) constitutes their most notable characteristic. This domain extends outside of the protein and leads to the formation of the firm bond of the serpin with its specific proteinase [18-20]. Members of the tick Serpin family have been studied and recommended as useful targets for tick vaccine development [21]. Consequently, serpin sequences from diverse tick species have been reported such as, *Amblyomma americanum* [22], *Amblyomma variegatum* [23]*, Amblyomma maculatum* [24], *Dermacentor variabilis* [25]; *Rhipicephalus appendiculatus* [26], *R. microplus* [6,27], *Haemaphysalis longicornis* [28], *Ixodes scapularis* [21,29], and *Ixodes ricinus* [9,11]. Additionally, an *in silico* identification of *R. microplus* serpin was conducted using different databases [30]. However, a great number of tick serpins continue to be functionally uncharacterised which limits the studies related with their function during host – parasite interaction [11,31,32].

In this study serpins from different *R. microplus* genomic databases were identified and four new serpins molecules were reported. *In silico* characterization of these serpins was undertaken using bioinformatics methods. Additionally, *R. microplus* serpins (RmS) were cloned, sequenced, and expressed in order to determine their protease inhibition specificity. The spatial expression of these serpins was carried out by PCR using cDNA from different tick life stages and female adult organs. Finally, this study is an important step forward in uncovering the role of RmS in the physiology of this ectoparasite and their potential use for the future improvement of ticks control methods.

## Methods

### Bioinformatics and Serpin identification

The identification of *R. microplus* serpin sequences was performed through a web-based bioinformatics environment called Yabi [33]. The current available tick serpin sequences of *Amblyomma americanum* [22], *A. maculatum* [24], *A. variegatum* [23], *A. monolakensis* [34] *H. longicornis* [28,35], *I. ricinus* [9,36], *Ixodes scapularis* [21], *R. microplus* [37], *R. appendiculatus* [26], and *A. monolakensis* [34] were retrieved from the National Centre for Biotechnology Information non-redundant protein (NCBI) (http://www.ncbi.nlm.nih.gov). These tick serpin sequences and the human α_1_-antitrypsin (GenBank, AAB59495) were used as queries against BmiGi V1 [38], BmiGi V2.1 [37], five SSH libraries [39], Australian tick transcriptome libraries [40] and RmiTR V1 [40] using the Basic Local Alignment Search Tool (BLAST) with the tblastX algorithm [41]. The qualified serpin sequences (E-value < 100) were six-frame translated for deduced protein sequences. The presence of the serpin conserved domain (cd00172) was analysed using the batch CD-Search Tool with an expected value threshold cut-off at 1 against NCBI’s Conserved Domain Database (CDD) [42]. SignalP 4.1 [43] was used to predict signal peptide cleavage sites. Also, the amino acid sequences of the *R. microplus* serpins were scanned for the presence of the C-terminal sequence Lys-Asp-Glu-Leu (KDEL) the endoplasmic reticulum lumen retention signal (KDEL motif, Prosite ID: PS00014) using ScanProSite (http://prosite.expasy.org/scanprosite/) in order to reduce the incidence of false positive results from the SignalP prediction. Putative N-glycosylation sites were predicted using the NetNGlyc 1.0 server (http://www.cbs.dtu.dk/services/NetNGlyc/).

### Tick sources

Hereford cattle at the tick colony maintained by Biosecurity Queensland from the Queensland Department of Agriculture, Fisheries and Forestry (DAFF) [44] were used to collect the acaricide susceptible strain *R. microplus* NRFS (Non-Resistant Field Strain). All of the eggs (E), larvae (L), nymphs (N), adult males (M) and feeding females (F) were collected from infested animals maintained within a moat pen (DAFF Animal Ethics approval SA2006/03/96). Tick organs were dissected from 17 day-old adult females for cDNA preparation including salivary glands (FSG), guts (FG) and ovaries (Ovr)*.*

### Total RNA extraction

RNA was isolated from eggs, nymphs, and the organs (guts, ovaries and salivary glands) dissected from semi-engorged females. Ticks/organs were ground in liquid nitrogen using diethylpyrocarbonate water-treated mortar and pestle prior to processing utilising the TRIzol® reagent (Gibco-BRL, USA). The tissue samples were stored in the ice-cold TRIzol® Reagent immediately after dissection, and then homogenised through a sterile 25-gauge needle. The total RNA was isolated following the manufacture’s protocol (Gibco-BRL, USA) and the mRNA was purified using Poly (A) Purist™ MAG Kit (Ambion, USA) as recommended by the manufacturer.

### Isolation, cloning and sequencing of rms genes

cDNA from nymphs, ovaries and salivary glands was synthesised from purified mRNA using the BioScript™ Kit (Clontech, USA) following the manufacturer’s recommended protocol. PCRs were conducted for isolation of the *rms* genes using gene specific 5′ and 3′ primers, and designed for the amplification of the coding sequences (CDS) of serpin. Following the amplification and confirmation of the PCR products by agarose gel electrophoresis, the PCR products were sub-cloned into the pCR 2.1-TOPO® vector following the manufacturer’s instructions (Invitrogen, USA). The recombinant plasmids obtained were named pCR-*rms*1, *rms2* and pCR-*rms* (n + 1). Ten individual colonies for each clone were selected and grown in 5 mL of LB broths supplemented with ampicillin (50 μg.mL^−1^) 18 hours prior to the purification of the plasmid using the QIAprep Spin miniprep kit (Qiagen, USA). The direct sequencing of the plasmid inserts was performed using the BigDye v3.1 technology (Applied Biosystems, USA) and analysed on the Applied Biosystems 3130xl Genetic Analyser at the Griffith University DNA Sequencing Facility (School of Biomolecular and Biomedical Science, Griffith University, Qld, Australia). The sequencing reactions were prepared using M13 primers in a 96-well plate format according to the manufacturer’s instructions (Applied Biosystems, USA). The sequences were visualised, edited and aligned using Sequencher v4.5 (Gene Codes Corporation, USA) to remove vector sequence and to thus confirm the CDS of the *rms* genes.

### Cloning and expression of RmS in the yeast P. pastoris

The coding sequence of *rms*1, -*rms3, -rms6,* and *rms15* were inserted into the pPICZα A and pPIC-B expression vector (Invitrogen, USA) for intracellular and extracellular expression. The resultant recombinant plasmids were transformed into the yeast *P. pastoris* GS115 and SMD1168H by electroporation as described in the *Pichia* Expression Kit manual (Invitrogen). The recombinant protein were purified from yeast pellet and supernatant using a Histrap FF 5 mL column (GE Healthcare, USA) as recommended by the manufacturer following by a gel filtration purification step using a HiLoad™ 16/600 Superdex™ 200 pg column (GE Healthcare, USA).

### Expression analysis by semi-quantitative Reverse Transcription (RT)-PCR

Gene specific primers were used to determine the gene expression pattern in eggs, nymphs, female guts, ovaries and salivary gland samples. A total of fifty ticks were dissected to isolate the different organs samples, and 25 nymphs were used on the preparation of the nymph sample. Approximately, five grams of eggs from ten different ticks were processed to conform this experimental sample. Briefly, the densitograms of amplified PCR products were analysed by ImageJ and normalised using the following formula, Y = V + V(H-X)X where Y = normalised mRNA density, V = observed *rms* PCR band density in individual samples, H = highest tick housekeeping gene PCR band density among tested samples, X = tick housekeeping gene density in individual samples [22]. All experimental samples were processed in triplicated.

### Protease inhibition assays

RmS-1, RmS-3, RmS*-*6 and RmS-15 expressed in *P. pastoris* yeast using the methodology reported previously [6,45] were used in this assay. The inhibition test was conducted as reported formerly [46] to screen the activity of RmS-1, -3, -6 and -15 against different proteases including bovine Chymotrypsin and Trypsin, porcine Elastase and Kallikrein, human Plasmin, and Thrombin (Sigma-Aldrich, USA). Briefly, 96-well plates were blocked with Blocking buffer (20 mM Tris-HCl, 150 mM NaCl and 5% skim milk, pH 7.6), and washed three times with Wash buffer (20 mM Tris-HCl, 150 mM NaCl, 0.01% Tween 20, pH 7.6) every 5 min. A total of 50 μL containing each protease were incubated with 50 fold molar of RmS-1, RmS-3, RmS*-*6 and RmS-15 at 37°C for 60 minutes in duplicate. The specific substrate (0.13 mM) was added and substrate hydrolysis was monitored every 30 second using Epoch Microplate Spectrophotometer (BioTek, USA) (see Table 1). The inhibition rate was calculated by comparing the enzymatic activity in the presence and absence of recombinant RmS. The experiments were conducted in triplicate.

**Table 1:** **The conditions of serpin inhibition reactions against commercially available bovine, porcine and human serine proteases**

Enzymes*	**[nM]**	**Binding buffer**	Substrates*	**[mM]**
Chymotrypsin	10	50 mM Tris-HCl, 150 mM NaCl, 20 mM CaCl_2_, 0.01 % Triton X-100, pH 8.0	N-Succinyl-Ala-Ala-Pro-Phe-*p*-nitroanilide	0.13
Elastase	50	50 mM Hepes, 100 mM Nacl, 0.01 % Triton X-100, pH 7.4	N-Succinyl-Ala-Ala-Ala-*p*-nitroanilide	0.13
Kallikrein	50	20 mM Tris-HCl, 150 mM NaCl, 0.02 % Triton X-100, pH 8.5	N-Benzoyl-Pro-Phe-Arg-*p*-nitroanilide hydrochloride	0.13
Plasmin	50	20 mM Tris-HCl, 150 mM NaCl, 0.02 % Triton X-100, pH 8.5	Gly-Arg-*p*-nitroanilide dihydrochloride	0.13
Thrombin	2	50 mM Tris-HCl, 150 mM NaCl, 20 mM CaCl_2_, 0.01 % Triton X-100, pH 8.0	Sar-Pro-Arg-*p*-nitroanilide dihydrochloride	0.13
Trypsin	2	50 mM Tris-HCl, 150 mM NaCl, 20 mM CaCl_2_, 0.01 % Triton X-100, pH 8.0,	N-Benzoyl-Phe-Val-Arg-*p*-nitroanilide hydrochloride	0.13

*All enzymes and substrates were purchased from Sigma-Aldrich, USA.

### Statistical analysis

The semi-quantitative PCR and protease inhibition assay data were evaluated by one-way ANOVA with Bonferroni testing (p ≤ 0.05). All analyses were conducted by the GraphPad Prism version 6.02 (GraphPad Software). Data were represented as the mean ± standard deviation (SD).

## Results

### Identification of RmS

The analysis of the *R. microplus* sequence databases revealed twenty-two different putative RmS ultimately identified after the elimination of redundant sequences. The full CDS for RmS-1 to RmS-18 reported by Tirloni and co-workers [30] were found in this study. The percentage identities after the alignment of the RmS was variable for example, RmS-3 and RmS-20 showed a 94% and 31% identities with hypothetical bacterial serpin (*Paraphysa parvula*) and *R. appendiculatus* Serpin-3, respectively. The reactive center loop characteristic of serine protease inhibitor family was found in the CDS of RmS- 19 to – 22. These new sequences were deposited in the Genbank with the following Accession Numbers: RmS-19: KP121409, RmS-20: KP121408, RmS-21: KP121411, and RmS-22: KP121414.

**Figure 1:**
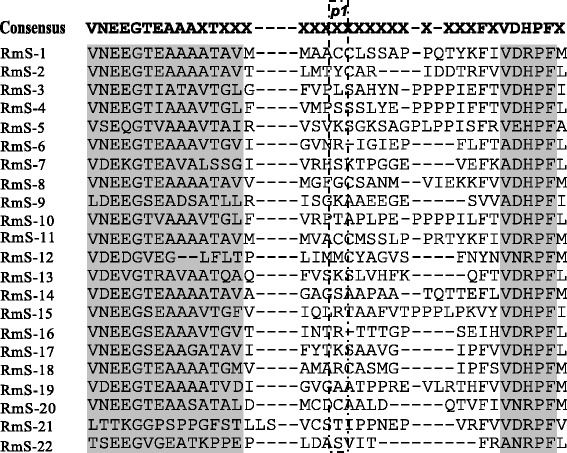
**Amino acid sequence alignment of the characteristic reactive center loops of** ***R. microplus*** **serpins (RmS-1 to –22).** Highly conserved residues and motifs were highlighted in gray shade. The P1 regions were highlighted with a dash dot line rectangle over the specific amino acid sequence [47].

There was observed a high variability of the identity among the RmS family that ranged from 29% between RmS-14 and RmS-15 to 62% between RmS-3 and RmS-5. The characteristic reactive center loop (RCL) domain associated with the serpin family members was found in all RmS (Figure 1). The type of amino acid at the P1 site of the RCL showed a high variation, for example in RmS-1, -4, 7, 10, 11, -14, 20 to 22 have a polar uncharged amino acid, but RmS-2, -3, -12 and -19 have hydrophobic amino acids. Basic amino acids such as arginine or lysine at the P1 site were found in RmS-5, -6, -9, -13. -15 to -18 (Figure 1). The consensus amino acid motif VNEEGT [47] and the canonical sequence representing the RCL hinge from P17 to P8 (EEGTIATAVT) [18] which are characteristic of serpins were highly conserved in the RmS aligned (Figure 1). Finally, the data confirmed the conservation of the reactive center loop and the characteristic motif of this proteins family in RmS -19 to RmS-22.

### RT-PCR analysis

**Figure 2:**
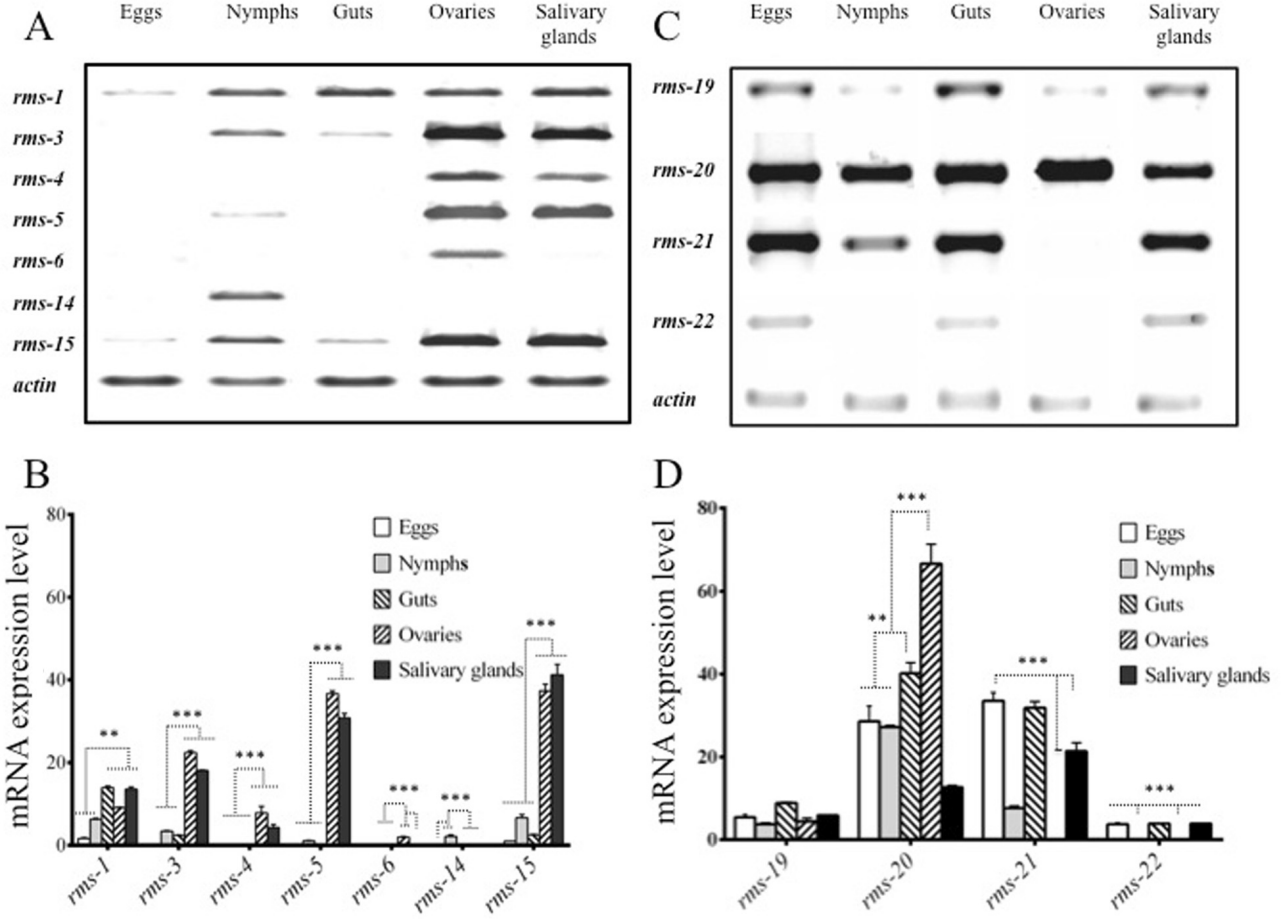
**Semi-quantitative analysis of the expression of** ***rms*** **transcripts in** ***R. microplus*** **tick samples** ***.*** **A** and **C**: PCR products obtained from cDNA samples from different tick’ tissues. The PCR products were run in 1% Tris Borate agarose gels. **B** and **D:** Normalised mRNA density was obtained after the densitogram analyses of the amplified PCR products. All experiments were conducted in triplicate. Data were represented as the mean ± standard deviation (SD). The symbols ** and *** indicate statistical significance with p < 0.05 and p < 0.001, respectively.

Reverse transcriptase PCR analysis was used to validate the spatial expression of *rms-1*, rms-3 to *-6*, *rms -14*, *rms -15, -19 to rms-22* transcripts. Data showed expression of the *rms* transcripts in different organs and developmental stages of *R. microplus* (Figure 2A -D). High expression of rms-1, -3, -5 and -15 in adult female compared with nymph stage was observed (Figure 2A and B), and *rms*-3 transcript was not detected in eggs. Expression of *rms -14* and *rms -6* was only detected in nymphs and ovaries respectively. The *rms -4* was detected only in ovaries and salivary glands (Figure 2A and B). The *rms*-1, -3, -5, *-15, -19* to *-22* transcripts were highly expressed in almost all tissues and tick stages analysed. The *rms-21* transcript was expressed in all tick samples except in ovaries, no expression of *rms-22* transcript was detected in nymph and ovaries (Figure 2C and D).

### Protease Inhibition of the recombinant R. microplus serpins (rRmS)

**Figure 3:**
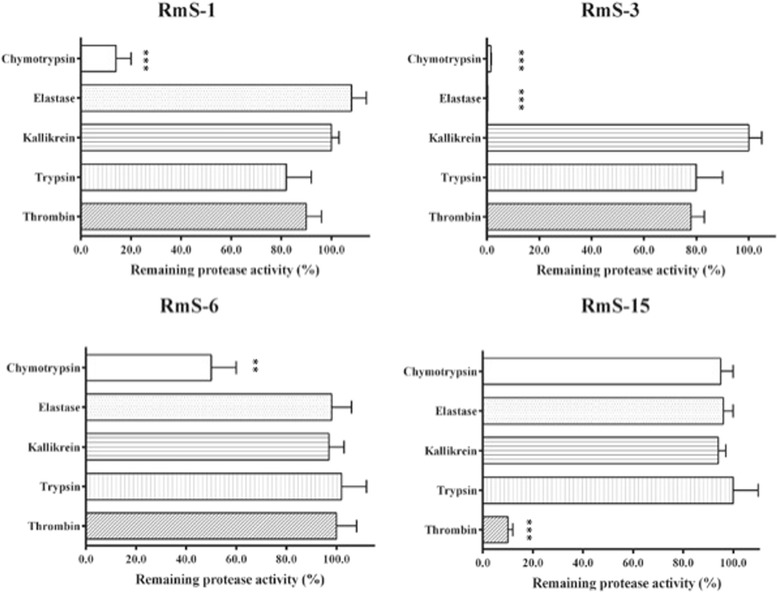
**Protease inhibition profiles for the recombinant RmS-1, -3, -6 and RmS-15 obtained from the yeast** ***P. pastoris*** **.** The RmS-1 and RmS-16 were expressed intracellular in the yeast *P. pastoris*. The symbols ** and *** indicate statistical significance with p < 0.05 and p < 0.001, respectively. The experiments were conducted in triplicate. Data were represented as the mean ± standard deviation (SD).

The coding sequences for *rms*-1, -3, -6 and -15 were cloned and expressed in *P. pastoris* yeast in order to test their inhibitory activity. These serpins were tested against different serine proteases including Chymotrypsin, Elastase, Kallikrein, Thrombin and Trypsin. The protease activity analysis showed that RmS-1 is a strong inhibitor of Chymotrypsin but weak inhibitor of Trypsin and Thrombin. RmS-3 has Chymotrypsin and Elastase as its principal target molecules and some faint inhibition of Trypsin and Thrombin was observed (Figure 3). RmS-15 exhibited strong inhibitory action of Thrombin while RmS-6 only inhibits Chymotrypsin (Figure 3).

## Discussion

The serpin family is conformed by a high and variable number of genes which are found in many different organisms, for example the human genome has approximately 36 serpins genes [48], 29 genes were found in *Drosophila melanogaster* [49], 45 in *I. scapularis*, and 17 serpins genes in *A. americanum* [21,22]. This corroborates a high conservation of the regulatory role of serpins among different species, and their functional versatility suggesting an evolutionary adaptation to confront different and novel proteases [50]. Processes such as host innate immune response regulation [51,52]; tick defences [49,53]; hemolymph coagulation cascade [54] and tick development [55,56] are regulated by serine protease inhibitors. In *ixodidae*, serpins are an extensive protein family with an important role at the physiological level, particularly during the parasitic periods of attachment and blood feeding [14,21-23,26,28,32,35,52,57,58]. Especially in *R. microplus*, a single host tick that has a highly efficient and complex combination of proteins in saliva that are useful in order to achieve the successful blood feeding, and serpins have an important role within tick saliva. In this study data obtained from transcriptome studies conducted on different stages of development of *R. microplus* and stored at CattleTick Base [40] were an important resource for this study to determine the members of the *R. microplus* serpin family [40]. However, full coverage of *R. microplus* genome would be necessary to give a precise number of *R. microplus* serpins [59]. Previous studies have provided important evidence of tick serpin sequences and transcript expression, but research discerning the specific targets or biological functions of these serpins is not forthcoming [6,13,32,52,60,61]. Following the elimination of redundant sequences the data obtained in this research suggested the presence of 22 putative *R. microplus* serpins from all databases studied. The amino acid sequences of these serpins revealed similar numbers of secreted and non-secreted serpins as described by Tirloni and co-workers [30]. A total of 18 *R. microplus* serpins showed high amino acid identity (range from 97 to 100%) with serpins reported in the BmGI and RmiTR V1 databases (including USA and Australian *R. microplus*), and those reported by Tirloni and co-worker (RmINCT-EM database, including Brazilian *R. microplus*) [30,40,62,63]. This observation confirms the conservation of these serpins in geographically distant populations of *R. microplus*.

The extracellular secretion of serine protease inhibitors during host – parasite interaction is important for ticks in order to overcome the haemostatic response of the host, blood digestion, and defence [64-70]. Anti-haemostasis serpins have been reported from *A. americanum* [32], *H. longicornis* [28,35], *I. ricinus* [9,11,57], and *R. haemaphysaloides* [71]. This study identified RmS-15 as an anti-haemostatic serpin that specifically inhibited Thrombin, an important serine protease of the coagulation pathway [72]. The result suggests an important role of RmS-15 to impair host blood coagulation during tick feeding. Similar results specifically related with blood coagulation was previously obtained for a mutant M340R of the *I. ricinus* serpin (Iris) that gained inhibitory activity against Thrombin and Factor Xa after losing its Elastase affinity through directed mutation [9].

This study improved the *P. pastoris* culture, expression and purification of previously described RmS-3 [6] demonstrated by significant inhibition of Chymotrypsin and Elastase observed in this study. The neutrophils’ elastase is discharged at the tick bite site which has reported to have an accumulation of this particular group of cells [73]. Additionally, previous studies have reported that neutrophils contribute to local inflammation during tick infestation which is an evasion mechanism employed by the host to resist tick infestation [67,70,74]. RmS-3 showed high levels of recognition by sera obtained from tick resistant cattle corroborating its secretion within tick saliva and an important role of this serpin during the host – parasite interaction [6]. RmS-3 might play an important role in the inhibition of host immune response. Similar results were obtained with the recombinant serpin from *I. ricinus* –Iris- with Elastase as its principal natural target [9,13]. However, the high expression of the *rms-3* gene observed in this study in tick ovaries is related with its possible role to protect tick reproductive cells from digestive proteases released into tick hemocoel. This defensive pathway was attributed to insect serpins that inhibit Chymotrypsin [75].

Serpins without a secretion signal have been reported to have a regulatory role in intracellular pathways such as tick development, intracellular digestion or vitellogenesis [67,68,76]. The predicted intracellular serpin, RmS-14 was only detected in nymphs showing specific expression of this serpin at this particular stage of tick development. RmS-14 was not detected by RT-PCR conducted previously in tissue samples from the Porto Alegro *R. microplus* strain (Rio Grande do Sur, Brazil) [30], however, nymph samples were not screened in this related study.

Four new serpins are reported in this investigation, two of them, RmS-19 and -20 were expressed in all tissues samples analysed showing their important role in both parasitic and non-parasitic stages of *R. microplus* development. RmS-21 and -22 were not detected in ovaries suggesting a regulatory role of these serpins in the proteolysis activity during digestion and embryos development in the eggs stage. Additionally, RmS-1 is a serpin that lacks a detectable signal peptide but was found to specifically inhibit Chymotrypsin with comparatively less inhibition of Trypsin and Thrombin. RmS-1 contains two methionines at P4, P5, and cysteines at P1 and P’1 sites of the RCL. The presence of these amino acids sensitive to oxidation (methionine and cysteine) at the RCL is characteristic of human intracellular serpins [77]. Also, RmS-1 clusters together with RAS1 and Lospin7, which are intracellular serpins from *R. appendiculatus* and *A. americanum* respectively [22,26]. The secreted and glycosylated RmS-1 expressed in *P. pastoris* had no significant inhibition against serine proteases tested in this study. However, protease inhibition data were obtained only using an intracellular and non­glycosylated RmS­1 expressed in *P. pastoris*. Data showed a significant inhibition of Chymotrypsin by the non­glycosylated RmS-1. The *rms-1* gene was expressed in all tissue samples analysed suggesting a broad regulatory role. Similar behaviour was observed with RmS-6, where only the intracellular and non­glycosylated RmS-6 showed activity against Chymotrypsin (Figure 3). The *rms16* was expressed only in the ovary sample suggesting a role for this serpin during tick embryogenesis or vitellogenesis. Further studies should be conducted in order to understand and characterise the activity and role during tick development and host parasite interaction of all *R. microplus* serpins identified.

## Conclusion

The present study provides an insight into the *R. microplus* serpin family allowing the study of differential expression within specific organs and different developmental stage with four new *R. microplus* serpins reported. The successful expression of recombinant serpins allowed the determination of their specific host target(s). Finally, the results obtained offer an important source of information to understand *R. microplus* serpin function and will deepen the knowledge about the role of serpins during tick-host interactions and tick development.
